# Principled missing data methods for researchers

**DOI:** 10.1186/2193-1801-2-222

**Published:** 2013-05-14

**Authors:** Yiran Dong, Chao-Ying Joanne Peng

**Affiliations:** Indiana University-Bloomington, Bloomington, Indiana USA

**Keywords:** Missing data, Listwise deletion, MI, FIML, EM, MAR, MCAR, MNAR

## Abstract

The impact of missing data on quantitative research can be serious, leading to biased estimates of parameters, loss of information, decreased statistical power, increased standard errors, and weakened generalizability of findings. In this paper, we discussed and demonstrated three principled missing data methods: multiple imputation, full information maximum likelihood, and expectation-maximization algorithm, applied to a real-world data set. Results were contrasted with those obtained from the complete data set and from the listwise deletion method. The relative merits of each method are noted, along with common features they share. The paper concludes with an emphasis on the importance of statistical assumptions, and recommendations for researchers. Quality of research will be enhanced if (a) researchers explicitly acknowledge missing data problems and the conditions under which they occurred, (b) principled methods are employed to handle missing data, and (c) the appropriate treatment of missing data is incorporated into review standards of manuscripts submitted for publication.

## Principled missing data methods for researchers

Missing data are a rule rather than an exception in quantitative research. Enders ( 2003 ) stated that a missing rate of 15% to 20% was common in educational and psychological studies. Peng et al. ( 2006 ) surveyed quantitative studies published from 1998 to 2004 in 11 education and psychology journals. They found that 36% of studies had no missing data, 48% had missing data, and about 16% cannot be determined. Among studies that showed evidence of missing data, 97% used the listwise deletion (LD) or the pairwise deletion (PD) method to deal with missing data. These two methods are ad hoc and notorious for biased and/or inefficient estimates in most situations ( Rubin 1987 ; Schafer 1997 ). The APA Task Force on Statistical Inference explicitly warned against their use ( Wilkinson and the Task Force on Statistical Inference 1999 p. 598). Newer and principled methods, such as the multiple-imputation (MI) method, the full information maximum likelihood (FIML) method, and the expectation-maximization (EM) method, take into consideration the conditions under which missing data occurred and provide better estimates for parameters than either LD or PD. Principled missing data methods do not replace a missing value directly; they combine available information from the observed data with statistical assumptions in order to estimate the population parameters and/or the missing data mechanism statistically.

A review of the quantitative studies published in *Journal of Educational Psychology* (JEP) between 2009 and 2010 revealed that, out of 68 articles that met our criteria for quantitative research, 46 (or 67.6%) articles explicitly acknowledged missing data, or were suspected to have some due to discrepancies between sample sizes and degrees of freedom. Eleven (or 16.2%) did not have missing data and the remaining 11 did not provide sufficient information to help us determine if missing data occurred. Of the 46 articles with missing data, 17 (or 37%) did not apply any method to deal with the missing data, 13 (or 28.3%) used LD or PD, 12 (or 26.1%) used FIML, four (or 8.7%) used EM, three (or 6.5%) used MI, and one (or 2.2%) used both the EM and the LD methods. Of the 29 articles that dealt with missing data, only two explained their rationale for using FIML and LD, respectively. One article misinterpreted FIML as an imputation method. Another was suspected to have used either LD or an imputation method to deal with attrition in a PISA data set ( OECD 2009 ; Williams and Williams 2010 ).

Compared with missing data treatments by articles published in JEP between 1998 and 2004 ( Table 3.1 in Peng et al. 2006 ), there has been improvement in the decreased use of LD (from 80.7% down to 21.7%) and PD (from 17.3% down to 6.5%), and an increased use of FIML (from 0% up to 26.1%), EM (from 1.0% up to 8.7%), or MI (from 0% up to 6.5%). Yet several research practices still prevailed from a decade ago, namely, not explicitly acknowledging the presence of missing data, not describing the particular approach used in dealing with missing data, and not testing assumptions associated with missing data methods. These findings suggest that researchers in educational psychology have not fully embraced principled missing data methods in research.

Although treating missing data is usually not the focus of a substantive study, failing to do so properly causes serious problems. First, missing data can introduce potential bias in parameter estimation and weaken the generalizability of the results ( Rubin 1987 ; Schafer 1997 ). Second, ignoring cases with missing data leads to the loss of information which in turn decreases statistical power and increases standard errors( Peng et al. 2006 ). Finally, most statistical procedures are designed for complete data ( Schafer and Graham 2002 ). Before a data set with missing values can be analyzed by these statistical procedures, it needs to be edited in some way into a “complete” data set. Failing to edit the data properly can make the data unsuitable for a statistical procedure and the statistical analyses vulnerable to violations of assumptions.

Because of the prevalence of the missing data problem and the threats it poses to statistical inferences, this paper is interested in promoting three principled methods, namely, MI, FIML, and EM, by illustrating these methods with an empirical data set and discussing issues surrounding their applications. Each method is demonstrated using SAS 9.3. Results are contrasted with those obtained from the complete data set and the LD method. The relative merits of each method are noted, along with common features they share. The paper concludes with an emphasis on assumptions associated with these principled methods and recommendations for researchers. The remainder of this paper is divided into the following sections: (1) Terminology, (2) Multiple Imputation (MI), (3) Full Information Maximum-Likelihood (FIML), (4) Expectation-Maximization (EM) Algorithm, (5) Demonstration, (6) Results, and (6) Discussion.

## Terminology

Missing data occur at two levels: at the unit level or at the item level. A unit-level non-response occurs when no information is collected from a respondent. For example, a respondent may refuse to take a survey, or does not show up for the survey. While the unit non-response is an important and common problem to tackle, it is not the focus of this paper. This paper focuses on the problem of *item non-response*. An item non-response refers to the incomplete information collected from a respondent. For example, a respondent may miss one or two questions on a survey, but answered the rest. The missing data problem at the item level needs to be tackled from three aspects: the proportion of missing data, the missing data mechanisms, and patterns of missing data. A researcher must address all three before choosing an appropriate procedure to deal with missing data. Each is discussed below.

### Proportion of missing data

The proportion of missing data is directly related to the quality of statistical inferences. Yet, there is no established cutoff from the literature regarding an acceptable percentage of missing data in a data set for valid statistical inferences. For example, Schafer ( 1999 ) asserted that a missing rate of 5% or less is inconsequential. Bennett ( 2001 ) maintained that statistical analysis is likely to be biased when more than 10% of data are missing. Furthermore, the amount of missing data is not the sole criterion by which a researcher assesses the missing data problem. Tabachnick and Fidell ( 2012 ) posited that the missing data mechanisms and the missing data patterns have greater impact on research results than does the proportion of missing data.

### Missing data mechanisms

According to Rubin ( 1976 ), there are three mechanisms under which missing data can occur: missing at random (MAR), missing completely at random (MCAR), and missing not at random (MNAR). To understand missing data mechanisms, we partition the data matrix *Y* into two parts: the observed part (*Y*_*obs*_) and the missing part (*Y*_*mis*_). Hence, *Y* = (*Y*_*obs*_, *Y*_*mis*_). Rubin ( 1976 ) defined MAR to be a condition in which the probability that data are missing depends only on the observed *Y*_*obs*_, but not on the missing *Y*_*mis*_, after controlling for *Y*_*obs*_. For example, suppose a researcher measures college students’ understanding of calculus in the beginning (pre-test) and at the end (post-test) of a calculus course. Let’s suppose that students who scored low on the pre-test are more likely to drop out of the course, hence, their scores on the post-test are missing. If we assume that the probability of missing the post-test depends only on scores on the pre-test, then the missing mechanism on the post-test is MAR. In other words, for students who have the same pre-test score, the probability of their missing the post-test is random. To state the definition of MAR formally, let *R* be a matrix of missingness with the same dimension as *Y*. The element of *R* is either 1 or 0, corresponding to *Y* being observed (coded as 1) or missing (coded as 0). If the distribution of *R*, written as *P*(*R*|*Y*, *ξ*), where ξ = missingness parameter, can be modeled as Equation 1, then the missing condition is said to be MAR ( Schafer 1997 p. 11):1

In other words, the probability of missingness depends on only the observed data and ξ. Furthermore, if (a) the missing data mechanism is MAR and (b) the parameter of the data model (*θ*) and the missingness parameter *ξ* are independent, the missing data mechanism is said to be ignorable ( Little and Rubin 2002 ). Since condition (b) is almost always true in real world settings, ignorability and MAR (together with MCAR) are sometimes viewed as equivalent ( Allison 2001 ).

Although many modern missing data methods (e.g., MI, FIML, EM) assume MAR, violation of this assumption should be expected in most cases ( Schafer and Graham 2002 ). Fortunately, research has shown that violation of the MAR assumption does not seriously distort parameter estimates ( Collins et al. 2001 ). Moreover, MAR is quite plausible when data are missing by design. Examples of missing by design include the use of multiple booklets in large scale assessment, longitudinal studies that measure a subsample at each time point, and latent variable analysis in which the latent variable is missing with a probability of 1, therefore, the missing probability is independent of all other variables.

MCAR is a special case of MAR. It is a missing data condition in which the likelihood of missingness depends neither on the observed data *Y*_*obs*_, nor on the missing data *Y*_*mis*_. Under this condition, the distribution of *R* is modeled as follows:2

If missing data meet the MCAR assumption, they can be viewed as a random sample of the complete data. Consequently, ignoring missing data under MCAR will not introduce bias, but will increase the *SE* of the sample estimates due to the reduced sample size. Thus, MCAR poses less threat to statistical inferences than MAR or MNAR.

The third missing data mechanism is MNAR. It occurs when the probability of missing depends on the missing value itself. For example, missing data on the income variable is likely to be MNAR, if high income earners are more inclined to withhold this information than average- or low-income earners. In case of MNAR, the missing mechanism must be specified by the researcher, and incorporated into data analysis in order to produce unbiased parameter estimates. This is a formidable task not required by MAR or MCAR.

The three missing data methods discussed in this paper are applicable under either the MCAR or the MAR condition, but not under MNAR. It is worth noting that including variables in the statistical inferential process that could explain missingness makes the MAR condition more plausible. Return to the college students’ achievement in a calculus course for example. If the researcher did not collect students’ achievement data on the pre-test, the missingness on the post-test is not MAR, because the missingness depends on the unobserved score on the post-test alone. Thus, the literature on missing data methods often suggests including additional variables into a statistical model in order to make the missing data mechanism ignorable ( Collins et al. 2001 ; Graham 2003 ; Rubin 1996 ).

The tenability of MCAR can be examined using Little’s multivariate test ( Little and Schenker 1995 ). However, it is impossible to test whether the MAR condition holds, given only the observed data ( Carpenter and Goldstein 2004 ; Horton and Kleinman 2007 ; White et al. 2011 ). One can instead examine the plausibility of MAR by a simple *t*-test of mean differences between the group with complete data and that with missing data ( Diggle et al. 1995 ; Tabachnick and Fidell 2012 ). Both approaches are illustrated with a data set at http://public.dhe.ibm.com/software/analytics/spss/documentation/statistics/20.0/en/client/Manuals/IBM_SPSS_Missing_Values.pdf. Yet, Schafer and Graham ( 2002 ) criticized the practice of dummy coding missing values, because such a practice redefines the parameters of the population. Readers should therefore be cautioned that the results of these tests should not be interpreted as providing definitive evidence of either MCAR or MAR.

### Patterns of missing data

There are three patterns of missing data: univariate, monotone, and arbitrary; each is discussed below. Suppose there are *p* variables, denoted as, *Y*_2_, *Y*_2_, …, *Y*_*p*_. A data set is said to have a univariate pattern of missing if the same participants have missing data on one or more of the *p* variables. A dataset is said to have a monotone missing data pattern if the variables can be arranged in such a way that, when *Y*_*j*_ is missing, *Y*_*j* + 1_, *Y*_*j* + 2_, …, *Y*_*p*_ are missing as well. The monotone missing data pattern occurs frequently in longitudinal studies where, if a participant drops out at one point, his/her data are missing on subsequent measures. For the treatment of missing data, the monotone missing data pattern subsumes the univariate missing data pattern. If missing data occur in any variable for any participant in a random fashion, the data set is said to have an arbitrary missing data pattern. Computationally, the univariate or the monotone missing data pattern is easier to handle than an arbitrary pattern.

## Multiple Imputation (MI)

MI is a principled missing data method that provides valid statistical inferences under the MAR condition ( Little and Rubin 2002 ). MI was proposed to impute missing data while acknowledging the uncertainty associated with the imputed values ( Little and Rubin 2002 ). Specifically, MI acknowledges the uncertainty by generating a set of *m* plausible values for each unobserved data point, resulting in *m* complete data sets, each with one unique estimate of the missing values. The *m* complete data sets are then analyzed individually using standard statistical procedures, resulting in *m* slightly different estimates for each parameter. At the final stage of MI, *m* estimates are pooled together to yield a single estimate of the parameter and its corresponding *SE*. The pooled *SE* of the parameter estimate incorporates the uncertainty due to the missing data treatment (the between imputation uncertainty) into the uncertainty inherent in any estimation method (the within imputation uncertainty). Consequently, the pooled *SE* is larger than the *SE* derived from a single imputation method (e.g., mean substitution) that does not consider the between imputation uncertainty. Thus, MI minimizes the bias in the *SE* of a parameter estimate derived from a single imputation method.

In sum, MI handles missing data in three steps: (1) imputes missing data *m* times to produce *m* complete data sets; (2) analyzes each data set using a standard statistical procedure; and (3) combines the *m* results into one using formulae from Rubin ( 1987 ) or Schafer ( 1997 ). Below we discuss each step in greater details and demonstrate MI with a real data set in the section **Demonstration**.

### Step 1: imputation

The imputation step in MI is the most complicated step among the three steps. The aim of the imputation step is to fill in missing values multiple times using the information contained in the observed data. Many imputation methods are available to serve this purpose. The preferred method is the one that matches the missing data pattern. Given a univariate or monotone missing data pattern, one can impute missing values using the regression method ( Rubin 1987 ), or the predictive mean matching method if the missing variable is continuous ( Heitjan and Little 1991 ; Schenker and Taylor 1996 ). When data are missing arbitrarily, one can use the Markov Chain Monte Carlo (MCMC) method ( Schafer 1997 ), or the fully conditional specification (also referred to as chained equations) if the missing variable is categorical or non-normal ( Raghunathan et al. 2001 ; van Buuren 2007 ; van Buuren et al. 1999 ; van Buuren et al. 2006 ). The regression method and the MCMC method are described next.

#### The regression method for univariate or monotone missing data pattern

Suppose that there are *p* variables, *Y*_1_, *Y*_2_, …, *Y*_*p*_ in a data set and missing data are uniformly or monotonically present from *Y*_*j*_ to *Y*_*p*_, where 1 <*j* ≤ *p*. To impute the missing values for the *j*th variable, one first constructs a regression model using observed data on *Y*_1_ through *Y*_*j* - 1_ to predict the missing values on *Y*_*j*_:3

The regression model in Equation 3 yields the estimated regression coefficients  and the corresponding covariance matrix. Based on these results, one can impute one set of regression coefficients  from the sampling distributions of . Next, the missing values in *Y*_*j*_ can be imputed by plugging  into Equation 3 and adding a random error. After missing data in *Y*_*j*_ are imputed, missing data in *Y*_*j* + 1_, …, *Y*_*p*_ are imputed subsequently in the same fashion, resulting in one complete data set. The above steps are repeated *m* times to derive *m* sets of missing values ( Rubin 1987 ; pp. 166–167; SAS Institute Inc 2011 ).

#### The MCMC method for arbitrary missing pattern

When the missing data pattern is arbitrary, it is difficult to develop analytical formulae for the missing data. One has to turn to numerical simulation methods, such as MCMC ( Schafer 1997 ) in this case. The MCMC technique used by the MI procedure of SAS is described below [interested readers should refer to SAS/STAT 9.3 User’s Guide ( SAS Institute Inc 2011 ) for a detailed explanation].

Recall that the goal of the imputation step is to draw random samples of missing data based on information contained in the observed data. Since the parameter (*θ*) of the data is also unknown, the imputation step actually draws random samples of both missing data and *θ* based on the observed data. Formally, the imputation step is to draw random samples from the distribution *P*(*θ*, *Y*_*mis*_|*Y*_*obs*_). Because it is much easier to draw estimates of *Y*_*mis*_ from *P*(*Y*_*mis*_|*Y*_*obs*_, *θ*) and estimates of *θ* from *P*(*θ*|*Y*_*obs*_, *Y*_*mis*_) separately, the MCMC method draws samples in two steps. At step one, given the current estimate of *θ*^(*t*)^ at the *t*th iteration, a random sample  is drawn from the conditional predictive distribution of *P*(*Y*_*mis*_|*Y*_*obs*_, *θ*^(*t*)^). At step two, given , a random sample of *θ*^(*t* + 1)^ is drawn from the distribution of . According to Tanner and Wong ( 1987 ), the first step is called the I-step (not to be confused with the first imputation step in MI) and the second step is called the P-step (or the posterior step). Starting with an initial value *θ*^(0)^ (usually an arbitrary guess), MCMC iterates between the I-step and the P-step, leading to a Markov Chain:  and so on. It can be shown that this Markov Chain converges in distribution to *P*(*θ*, *Y*_*mis*_|*Y*_*obs*_). It follows that the sequence *θ*^(1)^, *θ*^(2)^, …, *θ*^(*t*)^, … converges to *P*(*θ*|*Y*_*obs*_) and the sequence  converges to *P*(*Y*_*mis*_|*Y*_*obs*_). Thus, after the Markov Chain converges, *m* draws of *Y*_*mis*_ can form *m* imputations for the missing data. In practice, the *m* draws are separated by several iterations to avoid correlations between successive draws. Computation formulae of *P*(*Y*_*mis*_|*Y*_*obs*_, *θ*) and *P*(*θ*|*Y*_*obs*_, *Y*_*mis*_) based on the multivariate normal distribution can be found in SAS/STAT 9.3 User’s Guide ( SAS Institute Inc 2011 ). At the end of the first step in MI, *m* sets of complete data are generated.

### Step 2: statistical analysis

The second step of MI analyzes the *m* sets of data separately using a statistical procedure of a researcher’s choice. At the end of the second step, *m* sets of parameter estimates are obtained from separate analyses of *m* data sets.

### Step 3: combining results

The third step of MI combines the *m* estimates into one. Rubin ( 1987 ) provided formulae for combining *m* point estimates and *SE*s for a single parameter estimate and its *SE*. Suppose  denotes the estimate of a parameter *Q*, (e.g., a regression coefficient) from the *i*th data set. Its corresponding estimated variance is denoted as . Then the pooled point estimate of *Q* is given by:4

The variance of  is the weighted sum of two variances: the within imputation variance () and the between imputation variance (*B*). Specifically, these three variances are computed as follows:567

In Equation 7, the () factor is an adjustment for the randomness associated with a finite number of imputations. Theoretically, estimates derived from MI with small *m* yield larger sampling variances than ML estimates (e.g., those derived from FIML), because the latter do not involve randomness caused by simulation.

The statistic  is approximately distributed as a *t* distribution. The degrees of freedom (*ν*_*m*_ or ) for this *t* distribution are calculated by Equations 8–10 ( Barnard and Rubin 1999 ):8910

In Equation 8, *r* is the *relative increase in variance* due to missing data. The *r* is defined as the adjusted between-imputation variance standardized by the within-imputation variance. In Equation 10, *gamma* = (1 + 1/*m*)*B*/*T*, and *ν*_0_ is the degrees of freedom if the data are complete.  is a correction of *ν*_*m*_, when *ν*_0_ is small and the missing rate is moderate ( SAS Institute Inc 2011 ).

According to Rubin ( 1987 ), the severity of missing data is measured by the *fraction of missing information* (), defined as:11

As the number of imputations increases to infinity,  is reduced to the ratio of the between-imputation variance over the total variance. In its limiting form,  can be interpreted as the proportion of total variance (or total uncertainty) that is attributable to the missing data ( Schafer 1999 ).

For multivariate parameter estimation, Rubin ( 1987 ) provided a method to combine several estimates into a vector or matrix. The pooling procedure is a multivariate version of Equations (4) through (7), which incorporates the estimates of covariances among parameters. Rubin’s method assumes that the fraction of missing information (i.e., ) is the same for all variables ( SAS Institute Inc 2011 ). To our knowledge, no published studies have examined whether this assumption is realistic with real data sets, or Rubin’s method is robust to violation of this assumption.

### MI related issues

When implementing MI, the researcher needs to be aware of several practical issues, such as, the multivariate normality assumption, the imputation model, the number of imputations, and the convergence of MCMC. Each is discussed below.

#### The multivariate normality assumption

The regression and MCMC methods implemented in statistical packages (e.g., SAS) assume multivariate normality for variables. It has been shown that MI based on the multivariate normal model can provide valid estimates even when this assumption is violated ( Demirtas et al. 2008; Schafer 1997,1999 ). Furthermore, this assumption is robust when the sample size is large and when the missing rate is low, although the definition for a large sample size or for a low rate of missing is not specified in the literature ( Schafer 1997 ).

When an imputation model contains categorical variables, one cannot use the regression method or MCMC directly. Techniques such as, logistic regression and discriminant function analysis, can substitute for the regression method, if the missing data pattern is monotonic or univariate. If the missing data pattern is arbitrary, MCMC based on other probability models (such as the joint distribution of normal and binary) can be used for imputation. The free MI software NORM developed by Schafer ( 1997 ) has two add-on modules—CAT and MIX—that deal with categorical data. Specifically, CAT imputes missing data for categorical variables, and MIX imputes missing data for a combination of categorical and continuous variables. Other software packages are also available for imputing missing values in categorical variables, such as the ICE module in Stata ( Royston 2004,2005,2007 ; Royston and White 2011 ), the mice package in R and S-Plus ( van Buuren and Groothuis-Oudshoorn 2011 ), and the IVEware ( Raghunathan et al. 2001 (Yucel ). Interested readers are referred to a special volume of the *Journal of Statistical Software*^2011^ ) for recent developments in MI software.

When researchers use statistical packages that impose a multivariate normal distribution assumption on categorical variables, a common practice is to impute missing values based on the multivariate normal model, then round the imputed value to the nearest integer or to the nearest plausible value. However, studies have shown that this naïve way of rounding would not provide desirable results for binary missing values ( Ake 2005 ; Allison 2005 ; Enders 2010 ). For example, Horton et al. ( 2003 ) showed analytically that rounding the imputed values led to biased estimates, whereas imputed values without rounding led to unbiased results. Bernaards et al. ( 2007 ) compared three approaches to rounding in binary missing values: (1) rounding the imputed value to the nearest plausible value, (2) randomly drawing from a Bernoulli trial using the imputed value, between 0 and 1, as the probability in the Bernoulli trial, and (3) using an adaptive rounding rule based on the normal approximation to the binomial distribution. Their results showed that the second method was the worst in estimating odds ratio, and the third method provided the best results. One merit of their study is that it is based on a real-world data set. However, other factors may influence the performance of the rounding strategies, such as the missing mechanism, the size of the model, distributions of the categorical variables. These factors are not within a researcher’s control. Additional research is needed to identify one or more good strategy in dealing with categorical variables in MI, when a multivariate normal-based software is used to perform MI.

Unfortunately, even less is known about the effect of rounding in MI, when imputing ordinal variables with three or more levels. It is possible that as the level of the categorical variable increases, the effect of rounding decreases. Again, studies are needed to further explore this issue.

#### The imputation model

MI requires two models: the imputation model used in step 1 and the analysis model used in step 2. Theoretically, MI assumes that the two models are the same. In practice, they can be different ( Schafer 1997 ). An appropriate imputation model is the key to the effectiveness of MI; it should have the following two properties.

First, an imputation model should include useful variables. Rubin ( 1996 ) recommends a liberal approach when deciding if a variable should be included in the imputation model. Schafer ( 1997 ) and van Buuren et al. ( 1999 ) recommended three kinds of variables to be included in an imputation model: (1) variables that are of theoretical interest, (2) variables that are associated with the missing mechanism, and (3) variables that are correlated with the variables with missing data. The latter two kinds of variables are sometimes referred to as auxiliary variables ( Collins et al. 2001 ). The first kind of variables is necessary, because omitting them will downward bias the relation between these variables and other variables in the imputation model. The second kind of variables makes the MAR assumption more plausible, because they account for the missing mechanism. The third kind of variables helps to estimate missing values more precisely. Thus, each kind of variables has a unique contribution to the MI procedure. However, including too many variables in an imputation model may inflate the variance of estimates, or lead to non-convergence. Thus, researchers should carefully select variables to be included into an imputation model. van Buuren et al. ( 1999 ) recommended not including auxiliary variables that have too many missing data. Enders ( 2010 ) suggested selecting auxiliary variables that have absolute correlations greater than .4 with variables with missing data.

Second, an imputation model should be general enough to capture the assumed structure of the data. If an imputation model is more restrictive, namely, making additional restrictions than an analysis model, one of two consequences may follow. One consequence is that the results are valid but the conclusions may be conservative (i.e., failing to reject the false null hypothesis), if the additional restrictions are true ( Schafer 1999 ). Another consequence is that the results are invalid because one or more of the restrictions is false ( Schafer 1999 ). For example, a restriction may restrict the relationship between a variable and other variables in the imputation model to be merely pairwise. Therefore, any interaction effect that involves at least three variables will be biased toward zero. To handle interactions properly in MI, Enders ( 2010 ) suggested that the imputation model include the product of the two variables if both are continuous. For categorical variables, Enders suggested performing MI separately for each subgroup defined by the combination of the levels of the categorical variables.

#### Number of imputations

The number of imputations needed in MI is a function of the rate of missing information in a data set. A data set with a large amount of missing information requires more imputations. Rubin ( 1987 ) provided a formula to compute the relative efficiency of imputing *m* times, instead of an infinite number of times: RE = [1+ /*m*]^-1^, where  is the fraction of missing information, defined in Equation 11.

However, methodologists have not agreed on the optimal number of imputations. Schafer and Olsen ( 1998 ) suggested that “in many applications, just 3–5 imputations are sufficient to obtain excellent results” (p. 548). Schafer and Graham ( 2002) were more conservative in asserting that 20 imputations are enough in many practical applications to remove noises from estimations. Graham et al. ( 2007 ) commented that RE should not be an important criterion when specifying *m*, because RE has little practical meaning. Other factors, such as, the *SE*, *p*-value, and statistical power, are more related to empirical research and should also be considered, in addition to RE. Graham et al. ( 2007 ) reported that statistical power decreased much faster than RE, as λ increases and/or *m* decreases. In an extreme case in which λ=.9 and *m* = 3, the power for MI was only .39, while the power of an equivalent FIML analysis was 0.78. Based on these results, Graham et al. ( 2007 ) provided a table for the number of imputations needed, given λ and an acceptable power falloff, such as 1%. They defined the power falloff as the percentage decrease in power, compared to an equivalent FIML analysis, or compared to *m* = 100. For example, to ensure a power falloff less than 1%, they recommended *m* = 20, 40, 100, or > 100 for a true λ =.1, .5, .7, or .9 respectively. Their recommended *m* is much larger than what is derived from the Rubin rule based on RE ( Rubin 1987 ). Unfortunately, Graham et al.’s study is limited to testing a small standardized regression coefficient (β = 0.0969) in a simple regression analysis. The power falloff of MI may be less severe when the true β is larger than 0.0969. At the present, the literature does not shed light on the performance of MI when the regression model is more complex than a simple regression model.

Recently, White et al. ( 2011 ) argued that in addition to relative efficiency and power, researchers should also consider Monte Carlo errors when specifying the optimal number of imputations. Monte Carlo error is defined as the standard deviation of the estimates (e.g. regression coefficients, test statistic, *p*-value) “across repeated runs of the same imputation procedure with the same data” ( White et al. 2011 p. 387). Monte Carlo error converges to zero as *m* increases. A small Monte Carlo error implies that results from a particular run of MI could be reproduced in the subsequent repetition of the MI analysis. White et al. also suggested that the number of imputations should be greater than or equal to the percentage of missing observations in order to ensure an adequate level of reproducibility. For studies that compare different statistical methods, the number of imputations should be even larger than the percentage of missing observations, usually between 100 and 1000, in order to control the Monte Carlo error ( Royston and White 2011 ).

It is clear from the above discussions that a simple recommendation for the number of imputations (e.g., *m* = 5) is inadequate. For data sets with a large amount of missing information, more than five imputations are necessary in order to maintain the power level and control the Monte Carlo error. A larger imputation model may require more imputations, compared to a smaller or simpler model. This is so because a large imputation model results in increased *SE*s, compared to a smaller or simpler model. Therefore, for a large model, additional imputations are needed to offset the increased *SE*s. Specific guidelines for choosing *m* await empirical research. In general, it is a good practice to specify a sufficient *m* to ensure the convergence of MI within a reasonable computation time.

#### Convergence of MCMC

The convergence of the Markov Chain is one of the determinants of the validity of the results obtained from MI. If the Markov Chain does not converge, the imputed values are not considered random samples from the posterior distribution of the missing data, given the observed data, i.e., *P*(*Y*_*mis*_|*Y*_*obs*_). Consequently, statistical results based on these imputed values are invalid. Unfortunately, the importance of assessing the convergence was rarely mentioned in articles that reviewed the theory and application of MCMC ( Schafer 1999 ; Schafer and Graham 2002 ; Schlomer et al. 2010 ; Sinharay et al. 2001 ). Because the convergence is defined in terms of both probability and procedures, it is complex and difficult to determine the convergence of MCMC ( Enders 2010 ). One way to roughly assess convergence is to visually examine the trace plot and the autocorrelation function plot; both are provided by SAS PROC MI ( SAS Institute Inc 2011 ). For a parameter *θ*, a trace plot is a plot of the number of iterations (*t*) against the value of *θ*^(*t*)^ on the vertical axis. If the MCMC converges, there is no indication of a systematic trend in the trace plot. The autocorrelation plot displays the autocorrelations between *θ*^(*t*)^s at lag *k* on the vertical axis against *k* on the horizontal axis. Ideally, the autocorrelation at any lag should not be statistically significantly different from zero. Since the convergence of a Markov Chain may be at different rates for different parameters, one needs to examine these two plots for each parameter. When there are many parameters, one can choose to examine the worst linear function ( or WLF, Schafer 1997 ). The WLF is a constructed statistic that converges more slowly than all other parameters in the MCMC method. Thus if the WLF converges, all parameters should have converged (see pp. 2–3 of the Appendix for an illustration of both plots for WLF, accessible from https://oncourse.iu.edu/access/content/user/peng/Appendix.Dong%2BPeng.Principled%20missing%20methods.current.pdf). Another way to assess the convergence of MCMC is to start the chain multiple times, each with a different initial value. If all the chains yield similar results, one can be confident that the algorithm has converged.

## Full information maximum-likelihood (FIML)

FIML is a model-based missing data method that is used frequently in structural equating modeling (SEM). In our review of the literature, 26.1% studies that had missing data used FIML to deal with missing data. Unlike MI, FIML does not impute any missing data. It estimates parameters directly using all the information that is already contained in the incomplete data set. The FIML approach was outlined by Hartley and Hocking ( 1971 ). As the name suggests, FIML obtains parameter estimates by maximizing the likelihood function of the incomplete data. Under the assumption of multivariate normality, the log likelihood function of each observation *i* is:12

where *x*_*i*_ is the vector of observed values for case *i*, *K*_*i*_ is a constant that is determined by the number of observed variables for case *i*, and *μ* and Σ are, respectively, the mean vector and the covariance matrix that are to be estimated ( Enders 2001 ). For example, if there are three variables (*X*_1_, *X*_2_, and *X*_3_)  in the model. Suppose for case *i*, *X*_1_ = 10 and *X*_2_ = 5, while *X*_3_ is missing. Then in the likelihood function for case *i* is:

The total sample log likelihood is the sum of the individual log likelihood across *n* cases. The standard ML algorithm is used to obtain the estimates of *μ* and Σ, and the corresponding *SE*s by maximizing the total sample log likelihood function.

As with MI, FIML also assumes MAR and multivariate normality for the joint distribution of all the variables. When the two assumptions are met, FIML is demonstrated to produce unbiased estimates ( Enders and Bandalos 2001 ) and valid model fit information ( Enders 2001 ). Furthermore, FIML is generally more efficient than other ad hoc missing data methods, such as LD ( Enders 2001 ). When the normality assumption was violated, Enders ( 2001 ) reported that (1) FIML provided unbiased estimates across different missing rates, sample sizes, and distribution shapes, as long as the missing mechanism was MCAR or MAR, but (2) FIML resulted in negatively biased *SE* estimates and an inflated model rejection rate (namely, rejecting fitted models too frequently). Thus, Enders recommended using correction methods, such as rescaled statistics and bootstrap, to correct the bias associated with nonnormality.

Because FIML assumes MAR, adding auxiliary variables to a fitted model is beneficial to data analysis in terms of bias and efficiency ( Graham 2003 ; Section titled The Imputation Model). Collins et al. ( 2001 ) showed that auxiliary variables are especially helpful when (1) missing rate is high (i.e., > 50%), and/or (2) the auxiliary variable is at least moderately correlated (i.e., Pearson’s *r* > .4) with either the variable containing missing data or the variable causing missingness. However, incorporating auxiliary variables into FIML is not as straightforward as it is with MI. Graham ( 2003 ) proposed the saturated correlates model to incorporate auxiliary variables into a substantive SEM model, without affecting the parameter estimates of the SEM model or its model fit index. Specifically, Graham suggested that, after the substantive SEM model is constructed, the auxiliary variables be added into the model according to the following rules: (a) all auxiliary variables are specified to be correlated with all exogenous manifest variables in the model; (b) all auxiliary variables are specified to be correlated with the residuals for all the manifest variables that are predicted; and (c) all auxiliary variables are specified to be correlated to each other. Afterwards, the saturated correlates model can be fitted to data by FIML to increase efficiency and decrease bias.

## Expectation-maximization (EM) algorithm

The EM algorithm is another maximum-likelihood based missing data method. As with FIML, the EM algorithm does not “fill in” missing data, but rather estimates the parameters directly by maximizing the complete data log likelihood function. It does so by iterating between the E step and the M step ( Dempster et al. 1977 ).

The E (expectation) step calculates the expectation of the log-likelihood function of the parameters, given data. Assuming a data set (*Y*) is partitioned into two parts: the observed part and the missing part, namely, *Y* = (*Y*_*obs*_, *Y*_*mis*_). The distribution of *Y* depending on the unknown parameter *θ* can be therefore written as:13

Equation 13 can be written as a likelihood function as Equation 14:14

where *c* is a constant relating to the missing data mechanism that can be ignored under the MAR assumption and the independence between model parameters and the missing mechanism parameters ( Schafer 1997 p. 12). Taking the log of both sides of Equation 14 yields the following:15

where *l*(*θ*|*Y*) = log *P*(*Y*|*θ*) is the complete-data log likelihood, *l*(*θ*|*Y*_*obs*_) is the observed-data log likelihood, log *c* is a constant, and *P*(*Y*_*mis*_|*Y*_*obs*_, *θ* (Schafer ) is the predictive distribution of the missing data, given *θ*^1997^ ). Since log *c* does not affect the estimation of *θ*, this term can be dropped in subsequent calculations.

Because *Y*_*mis*_ is unknown, the complete-data log likelihood cannot be determined directly. However, if there is a temporary or initial guess of *θ* (denoted as *θ*^(*t*)^), it is possible to compute the expectation of *l*(*θ*|*Y*) with respect to the assumed distribution of the missing data *P*(*Y*_*mis*_|*Y*_*obs*_, *θ*^(*t*)^) as Equation 16:16

It is at the E step of the EM algorithm that *Q*(*θ*|*θ*^(*t*)^) is calculated.

At the M (Maximization) step, the next guess of *θ* is obtained by maximizing the expectation of the complete data log likelihood from the previous E step:17

The EM algorithm is initialized with an arbitrary guess of *θ*^0^, usually estimates based solely on the observed data. It proceeds by alternating between the E step and M step. It is terminated when successive estimates of *θ* are nearly identical. The *θ*^(*t*+1)^ that maximizes *Q*(*θ*|*θ*^(*t*)^) is guaranteed to yield an observed data log likelihood that is greater than or equal to that provided by *θ*^(*t*)^ ( Dempster et al. 1977 ).

The EM algorithm has many attractive properties. First, an EM estimator is unbiased and efficient when the missing mechanism is ignorable (ignorability is discussed under the section **Missing Data Mechanisms**, Graham 2003 ). Second, the EM algorithm is simple, easy to implement ( Dempster et al. 1977 ) and stable ( Couvreur 1996 ). Third, it is straightforward in EM to compare different models using the likelihood ratio test, because EM is based on the likelihood function. Assuming Model B is nested within Model A, these two models can be compared based on the difference in the log likelihoods corresponding to these two models, namely  Such a difference in the log likelihoods follows a chi-square distribution under suitable regularity conditions ( Schafer and Graham 2002 ; Wilks 1938 ). The degree of freedom of the chi-square statistic is the difference in the number of parameters estimated between the two models. Fourth, EM can be used in situations that are not missing data related. For example, EM algorithm can be used in mixture models, random effect models, mixed models, hierarchical linear models, and unbalanced designs including repeated measures ( Peng et al. 2006 ). Finally, the EM algorithm and other missing data methods that are based on the observed data log likelihood, such as FIML, are more efficient than the MI method because these methods do not require simulations whereas MI does.

However, the EM algorithm also has several disadvantages. First, the EM algorithm does not compute the derivatives of the log likelihood function. Consequently, it does not provide estimates of *SE*s. Although extensions of EM have been proposed to allow for the estimation of *SE*s, these extensions are computationally complex. Thus, EM is not a choice of the missing data method when statistical tests or confidence intervals of estimated parameters are the primary goals of research. Second, the rate of convergence can be painfully slow, when the percent of missing information is large ( Little and Rubin 2002 ). Third, many statistical programs assume the multivariate normal distribution when constructing *l*(*θ*|*Y*). Violation of this multivariate normality assumption may cause convergence problems for EM, and also for other ML-based methods, such as FIML. For example, if the likelihood function has more than one mode, the mode to which EM will converge depends on the starting value of the iteration. Schafer ( 1997 ) cautions that multiple modes do occur in real data sets, especially when “the data are sparse and/or the missingness pattern is unusually pernicious.” (p. 52). One way to check if the EM provides valid results is to initialize the EM algorithm with different starting values, and check if the results are similar. Finally, EM is model specific. Each proposed data model requires a unique likelihood function. In sum, if used flexibly and with *df*, EM is powerful and can provide smaller *SE* estimates than MI. Schafer and Graham ( 2002 ) compiled a list of packages that offered the EM algorithm. To the best of our knowledge, the list has not been updated in the literature.

## Demonstration

In this section, we demonstrate the three principled missing data methods by applying them to a real-world data set. The data set is complete and described under **Data Set**. A research question posted to this data set and an appropriate analysis strategy are described next under **Statistical Modeling**. From the complete data set, two missing data conditions were created under the MAR assumption at three missing data rates. These missing data conditions are described under **Generating Missing Data Conditions**. For each missing data condition, LD, MI, FIML, and EM were applied to answer the research question. The application of these four methods is described under **Data Analysis**. Results obtained from these methods were contrasted with those obtained from the complete data set. The results are discussed in the next section titled **Results**.

### Data Set

Self-reported health data by 432 adolescents were collected in the fall of 1988 from two junior high schools (Grades 7 through 9) in the Chicago area. Of the 432 participants, 83.4% were Whites and the remaining Blacks or others, with a mean age of 13.9 years and nearly even numbers of girls (*n* = 208) and boys (*n* = 224). Parents were notified by mail that the survey was to be conducted. Both the parents and the students were assured of their rights to optional participation and confidentiality of students’ responses. Written parental consent was waived with the approval of the school administration and the university Institutional Review Board ( Ingersoll et al. 1993 ). The adolescents reported their health behavior, using the Health Behavior Questionnaire (HBQ) ( Ingersoll and Orr 1989 ; Peng et al. 2006 ; Resnick et al. 1993 ), self-esteem, using Rosenberg’s inventory ( Rosenberg 1989 ), gender, race, intention to drop out of school, and family structure. The HBQ asked adolescents to indicate whether they engaged in specific risky health behaviors (Behavioral Risk Scale) or had experienced selected emotions (Emotional Risk Scale). The response scale ranged from 1 (*never*) to 4 (*about once a week*) for both scales. Examples of behavioral risk items were “I use alcohol (beer, wine, booze),” “I use pot,” and “I have had sexual intercourse/gone all the way.” These items measured frequency of adolescents’ alcohol and drug use, sexual activity, and delinquent behavior. Examples of emotional risk items were “I have attempted suicide,” and “I have felt depressed.” Emotional risk items measured adolescents’ quality of relationship with others, and management of emotions. Cronbach’s alpha reliability ( Nunnally 1978 ) was .84 for the Behavioral Risk Scale and .81 for the Emotional Risk Scale ( Peng and Nichols 2003 ). Adolescents’ self-esteem was assessed using Rosenberg’s self-esteem inventory ( Rosenberg 1989 ). Self-esteem scores ranged from 9.79 to 73.87 with a mean of 50.29 and *SD* of 10.04. Furthermore, among the 432 adolescents, 12.27% (*n* = 53) indicated an intention to drop out of school; 67.4% (*n* = 291) were from families with two parents, including those with one step-parent, and 32.63% (*n* = 141) were from families headed by a single parent. The data set is hereafter referred to as the ***Adolescent*** data and is available from https://oncourse.iu.edu/access/content/user/peng/logregdata_peng_.sav as an SPSS data file.

### Statistical Modeling

For the ***Adolescent*** data, we were interested in predicting adolescents’ behavioral risk from their gender, intention to drop out from school, family structure, and self-esteem scores. Given this objective, a linear regression model was fit to the data using adolescents’ score on the Behavioral Risk Scale of the HBQ as the dependent variable (BEHRISK) and gender (GENDER), intention to drop out of school (DROPOUT), type of family structure (FAMSTR), and self-esteem score (ESTEEM) as predictors or covariates. The emotional risk (EMORISK) was used subsequently as an auxiliary variable to illustrate the missing data methods. Hence, it was not included in the regression model. For the linear regression model, gender was coded as 1 for girls and 0 for boys, DROPOUT was coded as 1 for yes and 0 for no, and FAMSTR was coded as 1 for single-parent families and 0 for intact or step families. BEHRISK and ESTEEM were coded using participant’s scores on these two scales. Because the distribution of BEHRISK was highly skewed, a natural log transformation was applied to BEHRISK to reduce its skewness from 2.248 to 1.563. The natural-log transformed BEHRISK (or LBEHRISK) and ESTEEM were standardized before being included in the regression model to facilitate the discussion of the impact of different missing data methods. Thus, the regression model fitted to the ***Adolescent*** data was:18

The regression coefficients obtained from SAS 9.3 using the complete data were:19

According to the results, when all other covariates were held as a constant, boys, adolescents with intention to drop out of school, those with low self-esteem scores, or adolescents from single-parent families, were more likely to engage in risky behaviors.

### Generating missing data conditions

The missing data on LBEHRISK and ESTEEM were created under the MAR mechanism. Specifically, the probability of missing data on LBEHRISK was made to depend on EMORISK. And the probability of missing data on ESTEEM depended on FAMSTR. Peugh and Enders ( 2004 ) reviewed missing data reported in 23 applied research journals, and found that “the proportion of missing cases per analysis ranged from less than 1% to approximately 67%” (p. 539). Peng, et al. ( 2006 ) reported missing rates ranging from 26% to 72% based on 1,666 studies published in 11 education and psychology journals. We thus designed our study to correspond to the wide spread of missing rates encountered by applied researchers. Specifically, we manipulated the overall missing rate at three levels: 20%, 40%, or 60% (see Table 1).We did not include lower missing rates such as, 10% or 5%, because we expected missing data methods to perform similarly and better at low missing rates than at high missing rates. Altogether we generated three missing data conditions using SPSS 20 (see the Appendix for SPSS syntax for generating missing data). Due to the difficulty in manipulating missing data in the outcome variable and the covariates, the actual overall missing rates could not be controlled exactly at 20% or 60%. They did closely approximate these pre-specified rates (see the description below).

According to Table 1, at the 20% overall missing rate, participants from a single-parent family had a probability of .20 of missing ESTEEM, while participants from a two-parent family (including the intact families and families with one step- and one biological parent) had a probability of .02 of missing scores on ESTEEM. As the overall missing rate increased from 20% to 40% or 60%, the probability of missing on ESTEEM likewise increased. Furthermore, the probability of missing in LBEHRISK was conditioned on the value of EMORISK. Specifically, at the 20% overall missing rate, if EMORISK was at or below the first quartile, the probability of LBEHRISK missing was .00 (Table 1). If EMORISK was between the first and the third quartiles, the probability of LBEHRISK missing was .10 and an EMORISK at or above the third quartile resulted in LBEHRISK missing with a probability of .30. When the overall missing rate increased to 40% or 60%, the probabilities of missing LBEHRISK increased accordingly.

After generating three data sets with different overall missing rates, the regression model in Equation 18 was fitted to each data set using four methods (i.e., LD, MI, FIML, and EM) to deal with missing data. Since missing on LBEHRISK depended on EMORISK, EMORISK was used as an auxiliary variable in MI, EM, and FIML methods. All analyses were performed using SAS 9.3. For simplicity, we describe the data analysis for one of the three data sets, namely, the condition with an overall missing rate of 20%. Other data sets were analyzed similarly. Results are presented in Tables 2 and 3.

### Data analysis

#### *The LD method*

The LD method was implemented as a default in PROC REG. To implement LD, we ran PROC REG without specifying any options regarding missing data method. The SAS system, by default, used cases with complete data to estimate the regression coefficients.

**Table 1:** **Probability of missing for LBEHRISK and ESTEEM at three missing rates**

Overall missing rate	Missing variable	FAMSTR		Missing variable	EMORISK		
		Single family	Intact/step family		≤ ***Q1***	Between ***Q1*** &***Q3***	≥ ***Q3***
20%	ESTEEM	.20	.02	LBEHRISK	.00	.10	.30
40%	ESTEEM	.40	.05	LBEHRISK	.10	.20	.60
60%	ESTEEM	.80	.10	LBEHRISK	.20	.40	.80

*Note*. *Q1* = first quartile, *Q3* = third quartile.

**Table 2:** **Regression Coefficients from Four Missing Data Methods**

	Complete data	LD	MI	FIML	EM
**(a) Overall missing rate = 20%**^**a**^					
GENDER	-0.434***	-0.412***	-0.414***	-0.421***	-0.421***
	(0.082)	(0.091)	(0.086)	(0.087)	(0.083)
DROPOUT	1.172***	1.237***	1.266***	1.263***	1.263***
	(0.125)	(0.142)	(0.132)	(0.132)	(0.126)
ESTEEM	-0.191***	-0.213***	-0.215***	-0.212***	-0.212***
	(0.041)	(0.046)	(0.044)	(0.044)	(0.041)
FAMSTR	0.367***	0.377***	0.365***	0.366***	0.366***
	(0.087)	(0.101)	(0.096)	(0.092)	(0.088)
Actual *N*	432	349	432	N/A	414
**(b) Overall missing rate = 60%**^**b**^					
GENDER	-0.434***	-0.39**	-0.414***	-0.413***	-0.413***
	(0.082)	(0.131)	(0.1)	(0.104)	(0.086)
DROPOUT	1.172***	1.557***	1.559***	1.532***	1.562***
	(0.125)	(0.209)	(0.17)	(0.158)	(0.131)
ESTEEM	-0.191***	-0.193**	-0.217***	-0.214**	-0.215***
	(0.041)	(0.065)	(0.063)	(0.06)	(0.043)
FAMSTR	0.367***	0.479*	0.302*	0.3**	0.3**
	(0.087)	(0.192)	(0.116)	(0.111)	(0.091)
Actual *N*	432	171	432	N/A	367

*Note*. Standard error estimates in parentheses. MI results were based on 60 imputations. FIML results were obtained with EMORISK as an auxiliary variable in the model.

^a^The actual overall missing rate was 19.21%. ^b^The actual overall missing rate was 60.42%.

* *p* < .05. ** *p* < .01. ***p < .001.

#### *The MI method*

The MI method was implemented using a combination of PROC MI (for imputation), PROC REG (for OLS regression analysis), and PROC MIANALYZE (for pooling in MI). According to White et al. ( 2011 ), the number of imputations should be at least equal to the percentage of missing observations. The largest missing rate in the present study was 60%. Thus, we decided to impute missing data 60 times before pooling estimates. The imputation model included all four covariates specified in Equation 18, the dependent variable (LBEHRISK), and EMORISK as an auxiliary. For PROC MI, MCMC was chosen as the imputation method because the missing data pattern was arbitrary. By default, PROC MI uses the EM estimates as starting values for the MCMC method. The iteration history of EM indicated that the algorithm converged rather quickly; it took four iterations to converge for the 20% overall missing rate. The convergence in MCMC was further inspected using the trace plot and the autocorrelation function plot for the worst linear function ( SAS Institute Inc 2011 ). The inspection of the trace plot did not identify any systematic trend, or any significant autocorrelation for lags greater than two in the autocorrelation function plot. We therefore concluded that the MCMC converged and the choice of 1000 as the number of burn-in and 200 as the number of iterations between imputations was adequate. The number of burn-in is the number of iterations before the first draw. It needs to be sufficiently large to ensure the convergence of MCMC. The fraction of missing information (*λ*) for each variable with missing data was estimated by PROC MI to be .11 for LBEHRISK and .10 for ESTEEM. These s would have resulted in 3% power falloff, compared to FIML, if only five imputations were used ( Graham et al. 2007 ). Instead, we specified 60 imputations based on White et al. ( 2011 )’s recommendation. The resulting 60 imputed data sets were used in steps 2 and 3 of MI.

**Table 3:** **Percentage of Bias in Estimates**

	LD	MI	FIML	EM
**(a) Overall missing rate = 20%**^**a**^				
GENDER	5.07	4.61	3.00	3.00
DROPOUT	5.55	8.02	7.76	7.76
ESTEEM	-11.52	-12.57	-10.99	-10.99
FAMSTR	2.72	-0.54	-0.27	-0.27
**(b) Overall missing rate = 60%**^**b**^				
GENDER	10.14	4.61	4.84	4.84
DROPOUT	32.85	33.02	30.72	33.28
ESTEEM	-1.05	-13.61	-12.04	-12.57
FAMSTR	30.52	-17.71	-18.26	-18.26

*Note*. Percentage of bias was calculated as the ratio of the difference between the incomplete data estimate and the complete data estimate divided by the complete data estimate.

^a^The actual overall missing rate was 19.21%. ^b^The actual overall missing rate was 60.42%.

The second step in MI was to fit the regression model in Equation 18 to each imputed data set using PROC REG (see the Appendix for the SAS syntax). At the end of PROC REG, 60 sets of estimates of regression coefficients and their variance-covariance matrices were output to the third and final step in MI, namely, to pool these 60 estimates into one set. PROC MIANALYZE was invoked to combine these estimates and their variances/covariances into one set using the pooling formula in Equations 4 to 7 ( Rubin 1987 ). By default, PROC MIANALYZE uses *ν*_*m*_, defined in Equation 9, for hypothesis testing. In order to specify the corrected degrees of freedom *ν*_m_^*^ (as defined in Equation 10) for testing, we specified the “EDF=427” option, because 427 was the degrees of freedom based on the complete data.

#### *The FIML method*

The FIML method was implemented using PROC CALIS which is designed for structural equation modeling. Beginning with SAS 9.22, the CALIS procedure has offered an option to analyze data using FIML in the presence of missing data. The FIML method in the CALIS procedure has a variety of applications in path analyses, regression models, factor analyses, and others, as these modeling techniques are considered special cases of structural equation modeling ( Yung and Zhang 2011 ). For the current study, two models were specified using PROC CALIS: an ordinary least squares regression model without the auxiliary variable EMORISK, and a saturated correlates model that included EMORISK. For the saturated correlates model, EMORISK was specified to be correlated with the four covariates (GENDER, DROPOUT, ESTEEM, and FAMSTR) and the residual for LBEHRISK. Graham ( 2003 ) has shown that by constructing the saturated correlates model this way, one can include an auxiliary variable in the SEM model without affecting parameter estimate(s), or the model fit index for the model of substantive interest, which is Equation 18 in the current study.

#### *The EM method*

The EM method was implemented using both PROC MI and PROC REG. As stated previously, the versatile PROC MI can be used for EM if the EM statement was specified. To include auxiliary variables in EM, one lists the auxiliary variables on the VAR statement of PROC MI (see the Appendix for the SAS syntax). The output data set of PROC MI with the EM specification is a data set containing the estimated variance-covariance matrix and the vector of means of all the variables listed on the VAR statement. The variance-covariance matrix and the means vector were subsequently input into PROC REG to be fitted by the regression model in Equation 18. In order to compute the *SE* for the estimated regression coefficients, we specified a nominal sample size that was the average of available cases among all the variables. We decided on this strategy based on findings by Truxillo ( 2005 ). Truxillo ( 2005 ) compared three strategies for specifying sample sizes for hypothesis testing in discriminant function analysis using EM results. The three strategies were: (a) the minimum column-wise *n* (i.e., the smallest number of available cases among all variables), (b) the average column-wise *n* (i.e., the mean number of available cases among all the variables), and (c) the minimum pairwise *n* (the smallest number of available cases for any pair of variables in a data set). He found that the average column-wise *n* approach produced results closest to the complete-data results. It is worth noting that Truxillo ( 2005 )’s study was limited to discriminant function analysis and three sample size specifications. Additional research is needed in order to determine the best strategy to specify a nominal sample size for other statistical procedures.

## Results

Results derived from the 40% missing rate exhibited patterns between those obtained at 20% and 60% missing rates. Hence, they are presented in the Appendix. Table 2 presents estimates of regression coefficients and *SE*s derived from LD, MI, FIML and EM for the 20% and 60% missing data conditions. Table 3 presents the percent of bias in parameter estimates by the four missing data methods. The percentage of bias was defined and calculated as the ratio of the difference between the incomplete data estimate and the complete data estimate, divided by the complete data estimate. Any percentage of bias larger than 10% is considered substantial in subsequent discussions. The complete data results are included in Table 2 as a benchmark to which the missing data results are contrasted. The regression model based on the complete data explained 28.4% of variance (i.e., *R*_*adj*_^2^) in LBEHRISK, RMSE = 0.846, and all four predictors were statistically significant at *p* < .001.

According to Table 2, at 20% overall missing rate, estimates derived from the four missing data methods were statistically significant at *p* < .001, the same significance level as the complete data results. LD consistently resulted in larger *SE*, compared to the three principled methods, or the complete data set. The bias in estimates was mostly under 10%, except for estimates of ESTEEM by all four missing data methods (Table 3). The three principled methods exhibited similar biases and estimated FAMSTR accurately.

When the overall missing rate was 60% (Table 2), estimates derived from the four missing data methods showed that all four covariates were statistically significant at least at *p* < .05. LD consistently resulted in larger *SE*, compared to the three principled methods, or the complete data set. All four methods resulted in substantial bias for three of the four covariates (Table 3). The three principled methods once again yielded similar biases, whereas bias from LD was similar to these three only for DROPOUT. Indeed, DROPOUT was least accurately estimated by all four methods. LD estimated ESTEEM most accurately and better than the three principled methods. The three principled methods estimated GENDER most accurately and their estimates for FAMSTR were better than LD’s. Differences in absolute bias due to these four methods for ESTEEM or GENDER were actually quite small.

Compared to the complete data result, the three principled methods slightly overestimated *SE*s (Table 2), but not as badly as LD. Among the three methods, *SE*s obtained from EM were closer to those based on the complete data, than MI or FIML. This finding is to be expected because MI incorporates into *SE* the uncertainty associated with plausible missing data estimates. And the literature consistently documented the superior power of EM, compared to MI ( Collins et al. 2001 ; Graham et al. 2007 ; Schafer and Graham 2002 ).

In general, the *SE* and the bias increased as the overall missing rate increased from 20% to 60%. One exception to this trend was the bias in ESTEEM estimated by LD; they decreased instead, although the two estimates differed by a mere .02.

## Discussion

During the last decade, the missing data treatments reported in JEP have shown much improvement in terms of decreased use of ad hoc methods (e.g., LD and PD) and increased use of principled methods (e.g., FIML, EM, and MI). Yet several research practices still persisted including, not explicitly acknowledging the presence of missing data, not describing the approach used in dealing with missing data, not testing assumptions assumed. In this paper, we promote three principled missing data methods (i.e., MI, FIML, and EM) by discussing their theoretical framework, implementation, assumptions, and computing issues. All three methods were illustrated with an empirical *Adolescent* data set using SAS 9.3. Their performances were evaluated under three conditions. These three conditions were created from three missing rates (20%, 40%, and 60%). Each incomplete data set was subsequently analyzed by a regression model to predict adolescents’ behavioral risk score using one of the three principled methods or LD. The performance of the four missing data methods was contrasted with that of the complete data set in terms of bias and *SE*.

Results showed that the three principled methods yielded similar estimates at both missing data rates. In comparison, LD consistently resulted in larger *SE*s for regression coefficients estimates. These findings are consistent with those reported in the literature and thus confirm the recommendations of the three principled methods ( Allison 2003 ; Horton and Lipsitz 2001 ; Kenward and Carpenter 2007 ; Peng et al. 2006 ; Peugh and Enders 2004 ; Schafer and Graham 2002 ). Under the three missing data conditions, MI, FIML, and EM yielded similar estimates and *SE*s. These results are consistent with missing data theory that argues that MI and ML-based methods (e.g., FIML and EM) are equivalent ( Collins et al. 2001 ; Graham et al. 2007 ; Schafer and Graham 2002 ). In terms of *SE*, ML-based methods outperformed MI by providing slightly smaller *SE*s. This finding is to be expected because ML-based methods do not involve any randomness whereas MI does. Below we elaborate on features shared by MI and ML-based methods, choice between these two types of methods, and extension of these methods to multilevel research contexts.

### Features shared by MI and ML-based methods

First of all, these methods are based on the likelihood function of *P*(*Y*_*obs*_, *θ*) = ∫ *P*(*Y*_*complete*_, *θ*)*dY*_*mis*_. Because this equation is valid under MAR ( Rubin 1976 ), all three principled methods are valid under the MAR assumption. The two ML-based methods work directly with the likelihood function, whereas MI takes the Bayesian approach by imposing a prior distribution on the likelihood function. As the sample size increases, the impact of the specific prior distribution diminishes. It has been shown that,

If the user of the ML procedure and the imputer use the same set of input data (same set of variables and observational units), if their models apply equivalent distributional assumptions to the variables and the relationships among them, if the sample size is large, and if the number of imputations, *M*, is sufficiently large, then the results from the ML and MI procedures will be essentially identical. ( Collins et al. 2001 p. 336)

In fact, the computational details of EM and MCMC (i.e., data augmentation) are very similar ( Schafer 1997 ).

Second, both the MI and the ML-based methods allow the estimation/imputation model to be different from the analysis model—the model of substantive interest. Although it is widely known that the imputation model can be different from the analysis model for MI, the fact that ML-based methods can incorporate auxiliary variables (such as, EMORISK) is rarely mentioned in the literature, except by Graham ( 2003 ). As previously discussed, Graham ( 2003 ) suggested using the saturated correlates model to incorporate auxiliary variables into SEM. However, this approach results in a rapidly expanding model with each additional auxiliary variable; consequently, the ML-based methods may not converge. In this case, MI is the preferred method, especially when one needs to incorporate a large number of auxiliary variables into the model of substantive interest.

Finally, most statistical packages that offer the EM, FIML and/or MI methods assume multivariate normality. Theory and experiments suggest that MI is more robust to violation of this distributional assumption than ML-based methods ( Schafer 1997 ). As discussed previously, violation of the multivariate normality assumption may cause convergence problems for ML-based methods. Yet MI can still provide satisfactory results in the presence of non-normality (refer to the section titled **MI Related Issues**). This is so because the posterior distribution in MI is approximated by a finite mixture of the normal distributions. MI therefore is able to capture non-normal features, such as, skewness or multiple modes ( Schafer 1999 ). At the present, the literature does not offer systematic comparisons of these two methods in terms of their sensitivity to the violation of the multivariate normality assumption.

### Choice between MI and ML-based methods

The choice between MI and ML-based methods is not easy. On the one hand, ML-based methods offer the advantage of likelihood ratio tests so that nested models can be compared. Even though Schafer ( 1997 ) provided a way to combine likelihood ratio test statistics in MI, no empirical studies have evaluated the performance of this pooled likelihood ratio test under various data conditions (e.g., missing mechanism, missing rate, number of imputations, model complexity). And this test has not been incorporated into popular statistical packages, such as, SAS, SPSS. ML-based methods, in general, produce slightly smaller *SE*s than MI ( Collins et al. 2001 ; Schafer and Graham 2002 ). Finally, ML-based methods have greater power than MI ( Graham et al. 2007 ), unless imputations were sufficiently large, such as 100 or more.

On the other hand, MI has a clear advantage over ML-based methods when dealing with categorical variables ( Peng and Zhu 2008 ). Another advantage of MI over ML-based methods is its computational simplicity ( Sinharay et al. 2001 ). Once missing data have been imputed, fitting multiple models to a single data set does not require the repeated application of MI. Yet it requires multiple applications of ML-based methods to fit different models to the same data. As stated earlier, it is easier to include auxiliary variable in MI than in ML-based methods. In this sense, MI is the preferred method, if one wants to employ an inclusive strategy to selecting auxiliary variables.

The choice also depends on the goal of the study. If the aim is exploratory, or if the data are prepared for a number of users who may analyze the data differently, MI is certainly better than a ML-based method. For these purposes, a data analyst needs to make sure that the imputation model is general enough to capture meaningful relationships in the data set. If, however, a researcher is clear about the parameters to be estimated, FIML or EM is a better choice because they do not introduce randomness due to imputation into the data, and are more efficient than MI.

An even better way to deal with missing data is to apply MI and EM jointly. In fact, the application of MI can be facilitated by utilizing EM estimates as starting values for the data augmentation algorithm ( Enders 2010 ). Furthermore, the number of EM iterations needed for convergence is a conservative estimate for the number of burn-ins needed in data augmentation of MI, because EM converges slower than MI.

### Extension of MI and ML-based methods to multilevel research contexts

Many problems in education and psychology are multilevel in nature, such as students nested within classroom, teachers nested within school districts, etc. To adequately address these problems, multilevel model have been recommended by methodologists. For an imputation method to yield valid results, the imputation model must contain the same structure as the data. In other words, the imputation model should be multilevel in order to impute for missing data in a multilevel context ( Carpenter and Goldstein 2004 ). There are several ways to extend MI to deal with missing data when there are two levels. If missing data occur only at level 1 and the number of level 2 units is low, standard MI can be used with minor adjustments. For example, for a random-intercept model, one can dummy-code the cluster membership variable and include the dummy variables into the imputation model. In the case of a random slope and random intercepts model, one needs to perform multiple imputation separately within each cluster ( Graham 2009 ). When the number of level 2 units is high, the procedure just described is cumbersome. In this instance, one may turn to specialized MI programs, such as, the PAN library in the S-Plus program ( Schafer 2001 ), the REALCON-IMPUTE software ( Carpenter et al. 2011 ), and the R package mlmmm ( Yucel 2007 ). Unfortunately, ML-based methods have been extended to multilevel models only when there are missing data on the dependent variable, but not on the covariates at any level, such as student’s age at level 1 or school’s SES at level 2 ( Enders 2010 ).

In this paper, we discuss and demonstrate three principled missing data methods that are applicable for a variety of research contexts in educational psychology. Before applying any of the principled methods, one should make every effort to prevent missing data from occurring. Toward this end, the missing data rate should be kept at minimum by designing and implementing data collection carefully. When missing data are inevitable, one needs to closely examine the missing data mechanism, missing rate, missing pattern, and the data distribution before deciding on a suitable missing data method. When implementing a missing data method, a researcher should be mindful of issues related to its proper implementation, such as, statistical assumptions, the specification of the imputation/estimation model, a suitable number of imputations, and criteria of convergence.

Quality of research will be enhanced if (a) researchers explicitly acknowledge missing data problems and the conditions under which they occurred, (b) principled methods are employed to handle missing data, and (c) the appropriate treatment of missing data is incorporated into review standards of manuscripts submitted for publication.
